# A cuboidal [Cu_4_(SO_4_)_4_] structure supported by β-picoline ligands

**DOI:** 10.1107/S2056989022000780

**Published:** 2022-01-28

**Authors:** Ava M. Park, James A. Golen, David R. Manke

**Affiliations:** a Portsmouth Abbey School, 285 Cory’s Lane, Portsmouth, RI, 02871, USA; b University of Massachusetts Dartmouth, 285 Old Westport Road, North Dartmouth, MA 02747, USA

**Keywords:** crystal structure, picoline, sulfate, transition metal, coordination chemistry, cobalt complexes

## Abstract

The solid-state structure of the cobalt–β-picoline–sulfate complex tetra-μ_3_-sulfato-tetra­kis­[bis­(3-methyl­pyridine)­cobalt(II)], [Co_4_(SO_4_)_4_(C_6_H_7_N)_8_], is reported. The tetra­meric cobalt cluster contains a cuboidal core comprised of four cobalt(II) cations and four sulfate anions at alternate cube vertices. The cobalt corners are each capped with two β-picoline ligands. The sulfate anions adopt a rare [3.2110] bridging motif, and the cuboidal cluster is unprecedented in coordination chemistry.

## Abstract 2

A cobalt sulfate complex supported by β-picoline ligands produces a unique cuboidal tetra­mer.

## Chemical context

For the past few years, our lab has examined the solid-state structures of first-row transition-metal–pyridine–sulfate complexes (Park *et al.*, 2019 ▸; Pham *et al.*, 2018 ▸; Roy *et al.*, 2018 ▸). Despite the first such compound being reported in 1886 (Jørgensen, 1886 ▸; Manke, 2021 ▸), the structures of only two had been described in the literature when we started exploring this class of compounds. A series of these structures including Fe, Co, Ni, and Zn, showed one-dimensional coordination polymers exhibiting sulfate dianions bridging in *μ*-sulfato-*κ* ^2^ *O*:*O*′ modes. Inter­estingly, by modifying growth conditions, cobalt demonstrated two additional crystalline forms with variation in the bridging mode of sulfate ions that was not observed for the other metals. We have also explored the structural chemistry of such complexes with substituted pyridines, including *γ*-picoline, which showed similar structural chemistry to that observed with the pyridine ligand (Pham *et al.*, 2019 ▸). When we looked at the reaction of cobalt sulfate with β-picoline, a unique structure was obtained, a tetra­mer exhibiting an unprecedented cuboidal Cu_4_(SO_4_)_4_ core, described herein. 
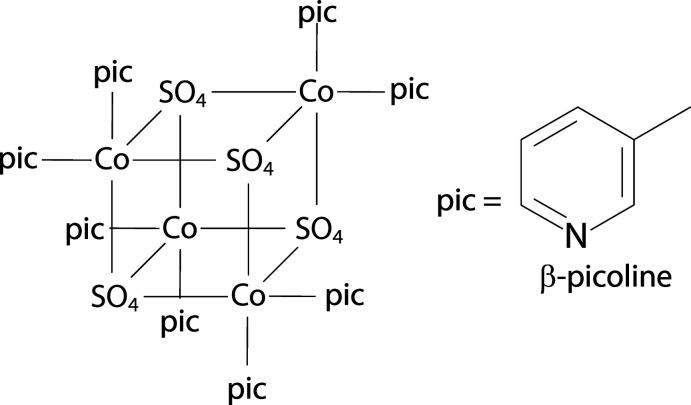


## Structural commentary

**Figure 1:**
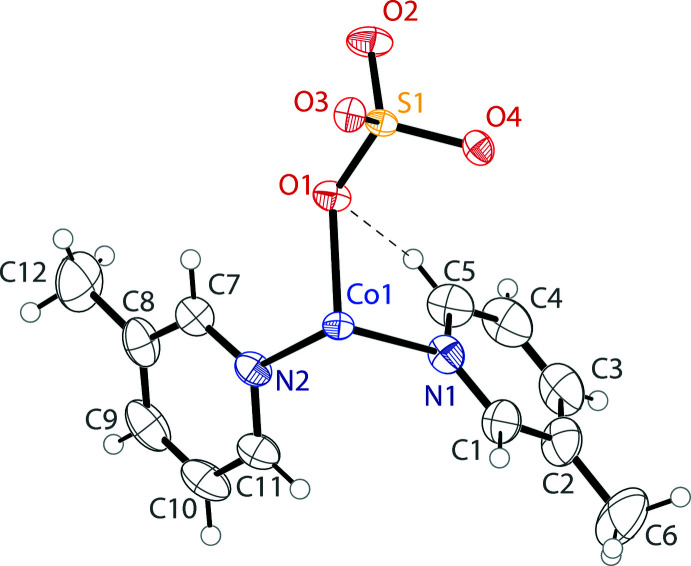
The asymmetric unit of the title compound showing the atomic labeling. Displacement ellipsoids are drawn at the 50% probability level. Hydrogen bonds are shown as dashed lines.

**Figure 2:**
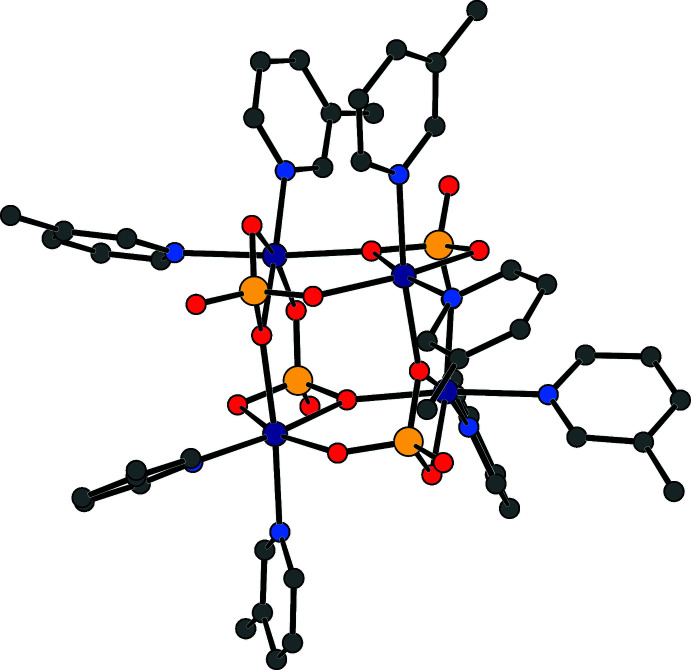
The [3.2110] coordination mode of sulfate in the title compound.

**Figure 3:**
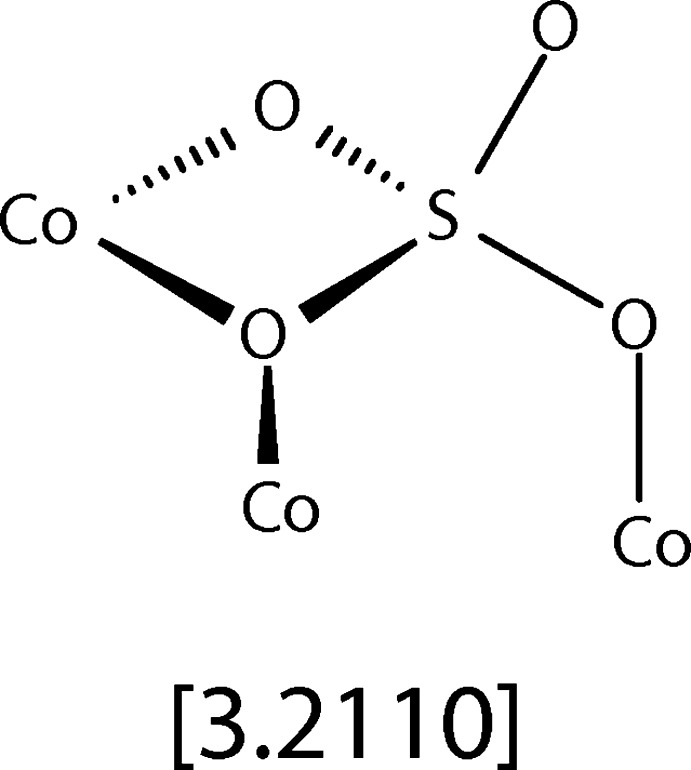
The cuboidal tetra­mer of the title compound. H atoms have been omitted for clarity.

The asymmetric unit of the title compound contains one cobalt cation, one sulfate anion, and two β-picoline ligands (Fig. 1 ▸). When grown out, the cobalt center demonstrates a pseudo-octa­hedral coordination environment. This consists of two β-picoline nitro­gen atoms, two oxygen atoms of a chelating sulfate ligand, one oxygen atom of a second sulfate anion, which bridges to another metal, and one terminal oxygen atom of a third sulfate ligand. The grown-out structure forms a tetra­mer of (β-pic)_2_CoSO_4_ units, demonstrating a cuboidal core in which four vertices are occupied by cobalt cations, and the other four vertices are occupied by sulfate anions (Fig. 2 ▸). The sulfate anions all bridge three Co^2+^ cations, demonstrating [3.2110] bridging by Harris notation (Fig. 3 ▸). Harris notation is written as [*X·YYYY*] where *X* is the number of metals that a ligand bridges, and the *Y*s are the number of metals connected to each donor atom in the ligand (Papatriantafyllopoulou *et al.*, 2009 ▸). The [3.2110] bridging motif is rare in sulfates and has only been observed in 1D coordination polymers of copper (Li *et al.*, 2008 ▸) and lanthanide/iron mixed-metal 3D coordination polymers (He *et al.*, 2017 ▸). There are two C—H⋯O inter­actions between the *ortho* hydrogens of one β-picoline ligand and the oxygens of two sulfate ions (Table 1 ▸). This results in a plane-to-plane angle between the CoN_3_O plane and the pyridine ring of 16.25 (9)°. These inter­actions are not present in the second unique picoline ligand, giving a larger plane-to-plane angle of 26.95 (9)°.

## Supra­molecular features

**Figure 4:**
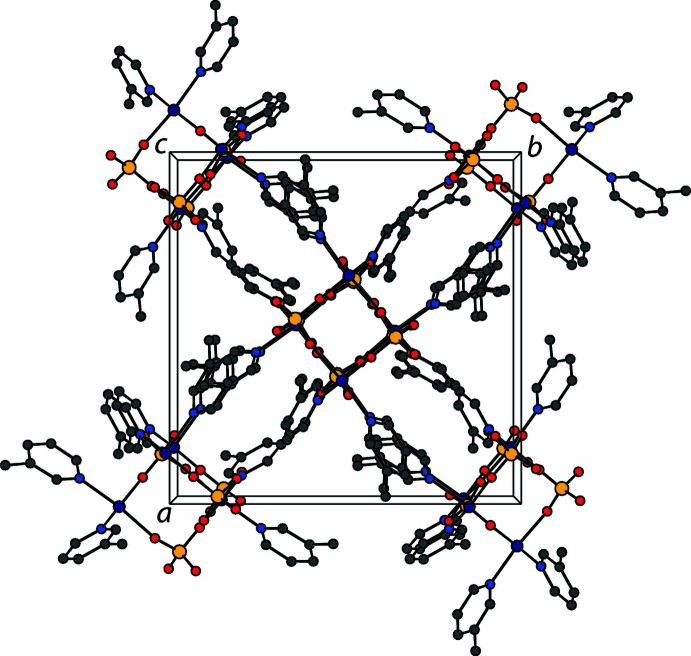
The crystal packing of the title compound shown along the *c* axis. H atoms have been omitted for clarity.

The crystal packing for the compound is shown in Fig. 4 ▸. The are weak C—H⋯O inter­actions between the *trans*-hydrogen atom of one picoline ligand and one of the terminal sulfate oxygens of a neighboring cuboid [C3—H3⋯O2^ii^; symmetry code: (ii) 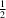 + *x*, 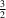 − *y*, 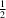 − *z*, Table 1 ▸). This inter­action might assist in the inter­digitation of the cuboids in the structure. No significant π–π inter­actions are observed.

## Database survey

The reported structures demonstrating sulfate ions with [3.2110] bridging modes are with copper (DOHKIV, DOHKIB: Li *et al.*, 2008 ▸) or mixtures of lanthanides with iron (He *et al.*, 2017 ▸), including dysprosium (DADNOO), erbium (DADPEG), europium (DADNII), gadolinium (DADNUU) and samarium (DADPAC). The prior structures of metal–pyridine sulfate complexes include three variations with pyridine (QIBFOZ: Pham *et al.*, 2018 ▸; QOXJAR, QOXJEV: Park *et al.*, 2019 ▸) and one with γ-picoline (ROFMIL: Pham *et al.*, 2019 ▸), all of which demonstrate 1D coordination polymers that are structurally quite different than the cuboidal compound reported here.

## Synthesis and crystallization

32 mg of CoSO_4_·7H_2_O were dissolved in 2.0 mL of 3-methyl­pyridine (Aldrich) and heated at 343 K for 24 h. Dark-pink crystals suitable for X-ray analysis were obtained.

## Refinement

Crystal data, data collection and structure refinement details are summarized in Table 2 ▸. Hydrogen atoms were placed in calculated positions [C—H = 0.93 Å (*sp* ^2^) and 0.96 Å (*sp* ^3^)]. Isotropic displacement parameters were set to 1.2*U*_eq_C (*sp* ^2^) or 1.5*U*_eq_C (*sp* ^3^).

## Supplementary Material

Crystal structure: contains datablock(s) I. DOI: 10.1107/S2056989022000780/zl5027sup1.cif

Structure factors: contains datablock(s) I. DOI: 10.1107/S2056989022000780/zl5027Isup2.hkl

CCDC reference: 2143864

Additional supporting information:  crystallographic information; 3D view; checkCIF report

## Figures and Tables

**Table 1:** Hydrogen-bond geometry (Å, °)

*D*—H⋯*A*	*D*—H	H⋯*A*	*D*⋯*A*	*D*—H⋯*A*
C1—H1⋯O4^i^	0.93	2.53	3.116 (4)	121
C3—H3⋯O2^ii^	0.93	2.46	3.135 (4)	129
C5—H5⋯O1	0.93	2.49	3.070 (4)	121

Symmetry codes: (i) y, -x+1, -z+1; (ii) x+{\script{1\over 2}}, -y+{\script{3\over 2}}, -z+{\script{1\over 2}}.

**Table 2:** Experimental details

Crystal data	
Chemical formula	[Co_4_(SO_4_)_4_(C_6_H_7_N)_8_]
*M*_r_	1364.96
Crystal system, space group	Tetragonal, *P*\overline{4}2_1_ *c*
Temperature (K)	298
*a*, *c* (Å)	15.6121 (16), 11.8359 (13)
*V* (Å^3^)	2884.9 (7)
*Z*	2
Radiation type	Mo *K*α
μ (mm^−1^)	1.35
Crystal size (mm)	0.24 × 0.22 × 0.20

Data collection	
Diffractometer	Bruker D8 Venture CMOS
Absorption correction	Multi-scan (*SADABS*; Bruker, 2018 ▸)
*T*_min_, *T*_max_	0.517, 0.562
No. of measured, independent and observed [*I* > 2σ(*I*)] reflections	54595, 2744, 2624
*R*_int_	0.037
(sin θ/λ)_max_ (Å^−1^)	0.611

Refinement	
*R*[*F* ^2^ > 2σ(*F* ^2^)], *wR*(*F* ^2^), *S*	0.019, 0.046, 1.14
No. of reflections	2744
No. of parameters	184
H-atom treatment	H-atom parameters constrained
Δρ_max_, Δρ_min_ (e Å^−3^)	0.16, −0.20
Absolute structure	Flack *x* determined using 1117 quotients [(*I* ^+^)−(*I* ^−^)]/[(*I* ^+^)+(*I* ^−^)] (Parsons et al., 2013 ▸)
Absolute structure parameter	0.007 (4)

Computer programs: *APEX3* and *SAINT* (Bruker, 2018 ▸), *SHELXT2014* (Sheldrick, 2015*a* ▸), *SHELXL2018* (Sheldrick, 2015*b* ▸), *OLEX2* (Dolomanov *et al.*, 2009 ▸), and *publCIF* (Westrip, 2010 ▸).

## supplementary crystallographic information

### Crystal data

**Table:**

[Co_4_(SO_4_)_4_(C_6_H_7_N)_8_]	*D*_x_ = 1.571 Mg m^−^^3^
*M**_r_* = 1364.96	Mo *K*α radiation, λ = 0.71073 Å
Tetragonal, *P*42_1_*c*	Cell parameters from 9496 reflections
*a* = 15.6121 (16) Å	θ = 2.9–25.7°
*c* = 11.8359 (13) Å	µ = 1.35 mm^−^^1^
*V* = 2884.9 (7) Å^3^	*T* = 298 K
*Z* = 2	BLOCK, pink
*F*(000) = 1400	0.24 × 0.22 × 0.20 mm

### Data collection

**Table:**

Bruker D8 Venture CMOS diffractometer	2624 reflections with *I* > 2σ(*I*)
φ and ω scans	*R*_int_ = 0.037
Absorption correction: multi-scan (SADABS; Bruker, 2018)	θ_max_ = 25.7°, θ_min_ = 2.9°
*T*_min_ = 0.517, *T*_max_ = 0.562	*h* = −19→19
54595 measured reflections	*k* = −19→19
2744 independent reflections	*l* = −14→14

### Refinement

**Table:**

Refinement on *F*^2^	H-atom parameters constrained
Least-squares matrix: full	*w* = 1/[σ^2^(*F*_o_^2^) + (0.0165*P*)^2^ + 1.2064*P*] where *P* = (*F*_o_^2^ + 2*F*_c_^2^)/3
*R*[*F*^2^ > 2σ(*F*^2^)] = 0.019	(Δ/σ)_max_ = 0.001
*wR*(*F*^2^) = 0.046	Δρ_max_ = 0.16 e Å^−^^3^
*S* = 1.14	Δρ_min_ = −0.20 e Å^−^^3^
2744 reflections	Extinction correction: SHELXL-2018/3 (Sheldrick 2015b), Fc^*^=kFc[1+0.001xFc^2^λ^3^/sin(2θ)]^-1/4^
184 parameters	Extinction coefficient: 0.0049 (4)
0 restraints	Absolute structure: Flack *x* determined using 1117 quotients [(*I*^+^)-(*I*^-^)]/[(*I*^+^)+(*I*^-^)] (Parsons et al., 2013)
Hydrogen site location: inferred from neighbouring sites	Absolute structure parameter: 0.007 (4)

### Special details

**Table:**

Geometry. All esds (except the esd in the dihedral angle between two l.s. planes) are estimated using the full covariance matrix. The cell esds are taken into account individually in the estimation of esds in distances, angles and torsion angles; correlations between esds in cell parameters are only used when they are defined by crystal symmetry. An approximate (isotropic) treatment of cell esds is used for estimating esds involving l.s. planes.

### Fractional atomic coordinates and isotropic or equivalent isotropic displacement parameters (Å^2^)

**Table:**

	*x*	*y*	*z*	*U*_iso_*/*U*_eq_	
Co1	0.64636 (2)	0.49053 (2)	0.41389 (3)	0.02066 (11)	
S1	0.52522 (4)	0.64004 (4)	0.35520 (5)	0.02043 (15)	
O1	0.58168 (12)	0.57205 (12)	0.30908 (15)	0.0267 (4)	
O2	0.49144 (15)	0.69145 (13)	0.26473 (17)	0.0370 (5)	
O3	0.45605 (11)	0.59894 (11)	0.42553 (16)	0.0240 (4)	
O4	0.57304 (12)	0.69234 (11)	0.43961 (15)	0.0258 (4)	
N1	0.75982 (14)	0.57112 (15)	0.4121 (2)	0.0319 (5)	
N2	0.70169 (16)	0.42286 (15)	0.2775 (2)	0.0293 (5)	
C1	0.82170 (19)	0.5691 (2)	0.4896 (3)	0.0400 (7)	
H1	0.813680	0.534627	0.552786	0.048*	
C2	0.8976 (2)	0.6156 (2)	0.4819 (3)	0.0489 (8)	
C3	0.9084 (2)	0.6660 (2)	0.3863 (3)	0.0546 (10)	
H3	0.958296	0.697697	0.376688	0.065*	
C4	0.8451 (2)	0.6690 (2)	0.3064 (3)	0.0541 (10)	
H4	0.851466	0.702977	0.242445	0.065*	
C5	0.7719 (2)	0.6210 (2)	0.3218 (3)	0.0434 (8)	
H5	0.729157	0.623471	0.267110	0.052*	
C6	0.9650 (3)	0.6110 (3)	0.5724 (4)	0.0829 (15)	
H6A	0.946445	0.572676	0.630913	0.124*	
H6B	0.973938	0.667009	0.603611	0.124*	
H6C	1.017567	0.590328	0.540358	0.124*	
C7	0.6638 (2)	0.4183 (2)	0.1768 (3)	0.0340 (7)	
H7	0.611734	0.446338	0.167140	0.041*	
C8	0.6978 (2)	0.3738 (2)	0.0853 (3)	0.0422 (7)	
C9	0.7741 (2)	0.3311 (2)	0.1032 (3)	0.0499 (9)	
H9	0.798504	0.299413	0.045000	0.060*	
C10	0.8143 (2)	0.3351 (2)	0.2062 (3)	0.0490 (9)	
H10	0.865907	0.306837	0.218114	0.059*	
C11	0.7766 (2)	0.3817 (2)	0.2914 (3)	0.0386 (8)	
H11	0.803962	0.384829	0.361126	0.046*	
C12	0.6512 (3)	0.3707 (3)	−0.0255 (3)	0.0732 (12)	
H12A	0.590895	0.377919	−0.012705	0.110*	
H12B	0.661205	0.316328	−0.061099	0.110*	
H12C	0.671634	0.415746	−0.073630	0.110*	

### Atomic displacement parameters (Å^2^)

**Table:**

	*U*^11^	*U*^22^	*U*^33^	*U*^12^	*U*^13^	*U*^23^
Co1	0.01998 (17)	0.02203 (18)	0.01997 (16)	0.00207 (14)	0.00180 (15)	0.00176 (14)
S1	0.0216 (3)	0.0201 (3)	0.0196 (3)	0.0031 (3)	0.0031 (2)	0.0046 (3)
O1	0.0293 (10)	0.0290 (10)	0.0218 (9)	0.0075 (8)	0.0056 (8)	0.0029 (8)
O2	0.0435 (12)	0.0380 (11)	0.0297 (10)	0.0114 (11)	−0.0007 (10)	0.0133 (9)
O3	0.0227 (9)	0.0272 (9)	0.0223 (9)	−0.0037 (7)	0.0042 (8)	0.0004 (8)
O4	0.0259 (9)	0.0213 (9)	0.0301 (11)	−0.0045 (8)	0.0048 (8)	0.0015 (7)
N1	0.0281 (12)	0.0299 (12)	0.0378 (13)	−0.0040 (10)	0.0080 (12)	0.0009 (12)
N2	0.0305 (12)	0.0272 (12)	0.0301 (13)	0.0033 (11)	0.0070 (11)	0.0008 (11)
C1	0.0349 (16)	0.0417 (17)	0.0434 (18)	−0.0091 (14)	0.0040 (14)	0.0014 (14)
C2	0.0337 (16)	0.051 (2)	0.062 (2)	−0.0128 (14)	0.0054 (17)	−0.0051 (19)
C3	0.0408 (19)	0.043 (2)	0.080 (3)	−0.0156 (16)	0.0191 (18)	0.0026 (18)
C4	0.052 (2)	0.042 (2)	0.068 (3)	−0.0086 (16)	0.019 (2)	0.0151 (18)
C5	0.0396 (18)	0.0437 (19)	0.0470 (19)	−0.0036 (15)	0.0103 (16)	0.0107 (16)
C6	0.048 (2)	0.102 (4)	0.098 (4)	−0.026 (2)	−0.017 (3)	0.002 (3)
C7	0.0376 (17)	0.0333 (15)	0.0310 (15)	−0.0005 (13)	0.0067 (13)	0.0015 (13)
C8	0.061 (2)	0.0340 (16)	0.0314 (15)	−0.0070 (14)	0.0115 (17)	−0.0043 (14)
C9	0.067 (2)	0.0349 (17)	0.048 (2)	0.0039 (16)	0.0277 (18)	−0.0072 (15)
C10	0.047 (2)	0.0364 (18)	0.063 (2)	0.0135 (15)	0.0187 (18)	−0.0001 (16)
C11	0.0369 (18)	0.0342 (17)	0.045 (2)	0.0085 (14)	0.0064 (14)	0.0007 (14)
C12	0.107 (4)	0.076 (3)	0.036 (2)	−0.002 (3)	−0.002 (3)	−0.012 (2)

### Geometric parameters (Å, º)

**Table:**

Co1—S1^i^	2.7458 (7)	C3—C4	1.368 (6)
Co1—O1	2.0441 (19)	C4—H4	0.9300
Co1—O3^i^	2.2037 (19)	C4—C5	1.379 (5)
Co1—O3^ii^	2.1274 (18)	C5—H5	0.9300
Co1—O4^i^	2.1229 (18)	C6—H6A	0.9600
Co1—N1	2.173 (2)	C6—H6B	0.9600
Co1—N2	2.114 (2)	C6—H6C	0.9600
S1—O1	1.4839 (19)	C7—H7	0.9300
S1—O2	1.438 (2)	C7—C8	1.391 (4)
S1—O3	1.5069 (18)	C8—C9	1.382 (5)
S1—O4	1.491 (2)	C8—C12	1.501 (5)
N1—C1	1.332 (4)	C9—H9	0.9300
N1—C5	1.336 (4)	C9—C10	1.371 (6)
N2—C7	1.333 (4)	C10—H10	0.9300
N2—C11	1.345 (4)	C10—C11	1.376 (5)
C1—H1	0.9300	C11—H11	0.9300
C1—C2	1.392 (4)	C12—H12A	0.9600
C2—C3	1.389 (5)	C12—H12B	0.9600
C2—C6	1.503 (5)	C12—H12C	0.9600
C3—H3	0.9300		
			
O1—Co1—S1^i^	129.94 (5)	N1—C1—H1	118.0
O1—Co1—O3^ii^	94.41 (7)	N1—C1—C2	124.0 (3)
O1—Co1—O3^i^	97.00 (7)	C2—C1—H1	118.0
O1—Co1—O4^i^	162.52 (7)	C1—C2—C6	121.6 (4)
O1—Co1—N1	92.08 (9)	C3—C2—C1	116.9 (3)
O1—Co1—N2	92.84 (9)	C3—C2—C6	121.5 (3)
O3^i^—Co1—S1^i^	33.21 (5)	C2—C3—H3	120.2
O3^ii^—Co1—S1^i^	81.36 (5)	C4—C3—C2	119.7 (3)
O3^ii^—Co1—O3^i^	86.57 (8)	C4—C3—H3	120.2
O3^ii^—Co1—N1	173.44 (9)	C3—C4—H4	120.4
O4^i^—Co1—S1^i^	32.58 (5)	C3—C4—C5	119.3 (3)
O4^i^—Co1—O3^ii^	83.94 (7)	C5—C4—H4	120.4
O4^i^—Co1—O3^i^	65.55 (7)	N1—C5—C4	122.6 (4)
O4^i^—Co1—N1	90.16 (9)	N1—C5—H5	118.7
N1—Co1—S1^i^	95.20 (8)	C4—C5—H5	118.7
N1—Co1—O3^i^	93.61 (8)	C2—C6—H6A	109.5
N2—Co1—S1^i^	136.90 (7)	C2—C6—H6B	109.5
N2—Co1—O3^i^	170.11 (9)	C2—C6—H6C	109.5
N2—Co1—O3^ii^	91.63 (8)	H6A—C6—H6B	109.5
N2—Co1—O4^i^	104.59 (8)	H6A—C6—H6C	109.5
N2—Co1—N1	87.07 (9)	H6B—C6—H6C	109.5
O1—S1—Co1^iii^	116.43 (7)	N2—C7—H7	118.2
O1—S1—O3	108.92 (10)	N2—C7—C8	123.6 (3)
O1—S1—O4	109.93 (11)	C8—C7—H7	118.2
O2—S1—Co1^iii^	133.48 (9)	C7—C8—C12	120.8 (3)
O2—S1—O1	110.07 (11)	C9—C8—C7	116.8 (3)
O2—S1—O3	112.71 (12)	C9—C8—C12	122.4 (3)
O2—S1—O4	112.15 (12)	C8—C9—H9	119.7
O3—S1—Co1^iii^	53.22 (7)	C10—C9—C8	120.6 (3)
O4—S1—Co1^iii^	50.06 (7)	C10—C9—H9	119.7
O4—S1—O3	102.82 (11)	C9—C10—H10	120.6
S1—O1—Co1	121.04 (10)	C9—C10—C11	118.7 (3)
Co1^ii^—O3—Co1^iii^	124.11 (9)	C11—C10—H10	120.6
S1—O3—Co1^ii^	141.48 (12)	N2—C11—C10	122.3 (3)
S1—O3—Co1^iii^	93.57 (9)	N2—C11—H11	118.9
S1—O4—Co1^iii^	97.36 (9)	C10—C11—H11	118.9
C1—N1—Co1	124.8 (2)	C8—C12—H12A	109.5
C1—N1—C5	117.5 (3)	C8—C12—H12B	109.5
C5—N1—Co1	117.4 (2)	C8—C12—H12C	109.5
C7—N2—Co1	121.9 (2)	H12A—C12—H12B	109.5
C7—N2—C11	118.1 (3)	H12A—C12—H12C	109.5
C11—N2—Co1	120.0 (2)	H12B—C12—H12C	109.5
			
Co1^iii^—S1—O1—Co1	−1.75 (15)	O4—S1—O3—Co1^iii^	−7.25 (10)
Co1^iii^—S1—O3—Co1^ii^	−168.6 (2)	N1—C1—C2—C3	−0.5 (5)
Co1—N1—C1—C2	173.9 (3)	N1—C1—C2—C6	179.7 (4)
Co1—N1—C5—C4	−174.0 (3)	N2—C7—C8—C9	1.5 (5)
Co1—N2—C7—C8	−179.4 (2)	N2—C7—C8—C12	179.5 (3)
Co1—N2—C11—C10	178.4 (2)	C1—N1—C5—C4	0.4 (5)
O1—S1—O3—Co1^iii^	109.34 (9)	C1—C2—C3—C4	0.8 (5)
O1—S1—O3—Co1^ii^	−59.3 (2)	C2—C3—C4—C5	−0.6 (6)
O1—S1—O4—Co1^iii^	−108.29 (10)	C3—C4—C5—N1	−0.1 (6)
O2—S1—O1—Co1	176.64 (13)	C5—N1—C1—C2	−0.1 (5)
O2—S1—O3—Co1^iii^	−128.20 (11)	C6—C2—C3—C4	−179.4 (4)
O2—S1—O3—Co1^ii^	63.1 (2)	C7—N2—C11—C10	−0.6 (4)
O2—S1—O4—Co1^iii^	128.92 (11)	C7—C8—C9—C10	−1.5 (5)
O3—S1—O1—Co1	−59.31 (15)	C8—C9—C10—C11	0.6 (5)
O3—S1—O4—Co1^iii^	7.57 (11)	C9—C10—C11—N2	0.5 (5)
O4—S1—O1—Co1	52.63 (15)	C11—N2—C7—C8	−0.5 (4)
O4—S1—O3—Co1^ii^	−175.90 (17)	C12—C8—C9—C10	−179.4 (3)

Symmetry codes: (i) *y*, −*x*+1, −*z*+1; (ii) −*x*+1, −*y*+1, *z*; (iii) −*y*+1, *x*, −*z*+1.

### Hydrogen-bond geometry (Å, º)

**Table:**

*D*—H···*A*	*D*—H	H···*A*	*D*···*A*	*D*—H···*A*
C1—H1···O4^i^	0.93	2.53	3.116 (4)	121
C3—H3···O2^iv^	0.93	2.46	3.135 (4)	129
C5—H5···O1	0.93	2.49	3.070 (4)	121

Symmetry codes: (i) *y*, −*x*+1, −*z*+1; (iv) *x*+1/2, −*y*+3/2, −*z*+1/2.
