# A cuboidal [Co_4_(SO_4_)_4_] structure supported by β-picoline ligands. Corrigendum

**DOI:** 10.1107/S2056989022002249

**Published:** 2022-03-01

**Authors:** Ava M. Park, James A. Golen, David R. Manke

**Affiliations:** a Portsmouth Abbey School, 285 Cory’s Lane, Portsmouth, RI, 02871, USA; b University of Massachusetts Dartmouth, 285 Old Westport Road, North Dartmouth, MA 02747, USA

## Abstract

In the paper by Park *et al.* [ *Acta Cryst.* (2022), E**78**, 108–110], there is an error in the chemical formula in the title.

## Abstract 2

Corrigendum to *Acta Cryst.* (2022), E**78**, 108–110.

The chemical formula in the title of the paper by Park *et al.* (2022 ▸) is incorrect as ‘[Cu_4_(SO_4_)_4_]’ and should be ‘[Co_4_(SO_4_)_4_]’, as given above.

## References

[bb1] Park, A. M., Golen, J. A. & Manke, D. R. (2022). *Acta Cryst.* E**78**, 108–110. 10.1107/S2056989022000780 PMC8819428 35145733

